# Dual-source Computed Tomography for Evaluating Pulmonary Artery and Aorta in Pediatric Patients with Single Ventricle

**DOI:** 10.1038/s41598-017-11809-6

**Published:** 2017-10-17

**Authors:** Meng-xi Yang, Zhi-gang Yang, Yi Zhang, Ke Shi, Hua-yan Xu, Kai-yue Diao, Ying-kun Guo

**Affiliations:** 1 State Key Laboratory of Biotherapy and Cancer Center, West China Hospital, Sichuan University, Chengdu, China; 2 Department of Radiology, West China Hospital, Sichuan University, Chengdu, China; 3 Department of Radiology, Key Laboratory of Obstetric & Gynecologic and Pediatric Diseases and Birth Defects of Ministry of Education, West China Second University Hospital, Sichuan University, Chengdu, China

## Abstract

To explore the accuracy of main pulmonary artery (MPA) and ascending aorta (AAO) image evaluation in pediatric patients with single ventricle (SV) by comparing dual-source computed tomography (DSCT) with echocardiography. Thirty-one children with SV were retrospectively enrolled. The stenosis, dilation, and location of MPA and AAO were independently evaluated by DSCT and echocardiography. The accompanying arterial malformations were also assessed by DSCT. For 17 patients undergoing cardiac catheterization, the DSCT-based diameters of MPA and AAO were correlated with their pressures as measured by catheterization. Referring to the surgical and catheterization findings, DSCT had better diagnostic performance in detecting the stenosis, dilation, and location of MPA and AAO with higher sensitivity than echocardiography (sensitivity, MPA: 88.0% vs. 80.0%, AAO: 100% vs. 66.7%, great arteries location: 95.7% vs. 95.2%). The correlations between diameters of MPA and AAO with their pressures were 0.399 (*p* = 0.04) and 0.611 (*p* = 0.01), respectively. In addition, DSCT detected 23 cases with patent ductus arteriosus, 26 systemic-to-pulmonary collaterals, 9 branch pulmonary distortions, and 4 coronary artery anomalies. DSCT is reliable for assessing the anatomic features of pulmonary artery and aorta in SV children, and provides comprehensive information for surgical strategy-making.

## Introduction

Single ventricle (SV) is a rare category of congenital heart disease (CHD) with a prevalence of 54 per million live births. It is characterized by both atria draining entirely, or almost entirely, into a single double-inlet ventricle^1^. Actually, SV is frequently associated with various cardiovascular lesions, especially anomalies of the great arteries. In clinical practice, the main interventional strategies for SV include palliative operations in three stages. The anatomy of the mediastinal great arteries and the hemodynamic states impact the surgical and interventional determinations, particularly in stage I surgery^1,2^. It is necessary to obtain a comprehensive evaluation of the pulmonary artery (PA) and aorta prior to surgical interventions.

Echocardiography, as the first-line choice for SV assessment, allows accurate evaluation of intra-cardiac anatomic details and cardiac function. However, the small acoustic window limits its efficacy in evaluating extra-cardiac vessels^3^. Recently, dual-source computed tomography (DSCT) has been widely applied in complex CHD owing to its fast scanning speed, low radiation dose, and high special resolution^4–6^. However, to the best of our knowledge, there is a lack of published data focusing on the reliability of DSCT in assessing extra-cardiac great arteries in patients with SV. Therefore, our study aims to explore the accuracy of main pulmonary artery (MPA) and ascending aorta (AAO) assessments using DSCT in comparison with trans-thoracic echocardiography (TTE), and to determine whether additional valuable information can be provided by DSCT for surgical planning and clinical decision-making.

## Results

### Baseline characteristics

**Figure 1:**
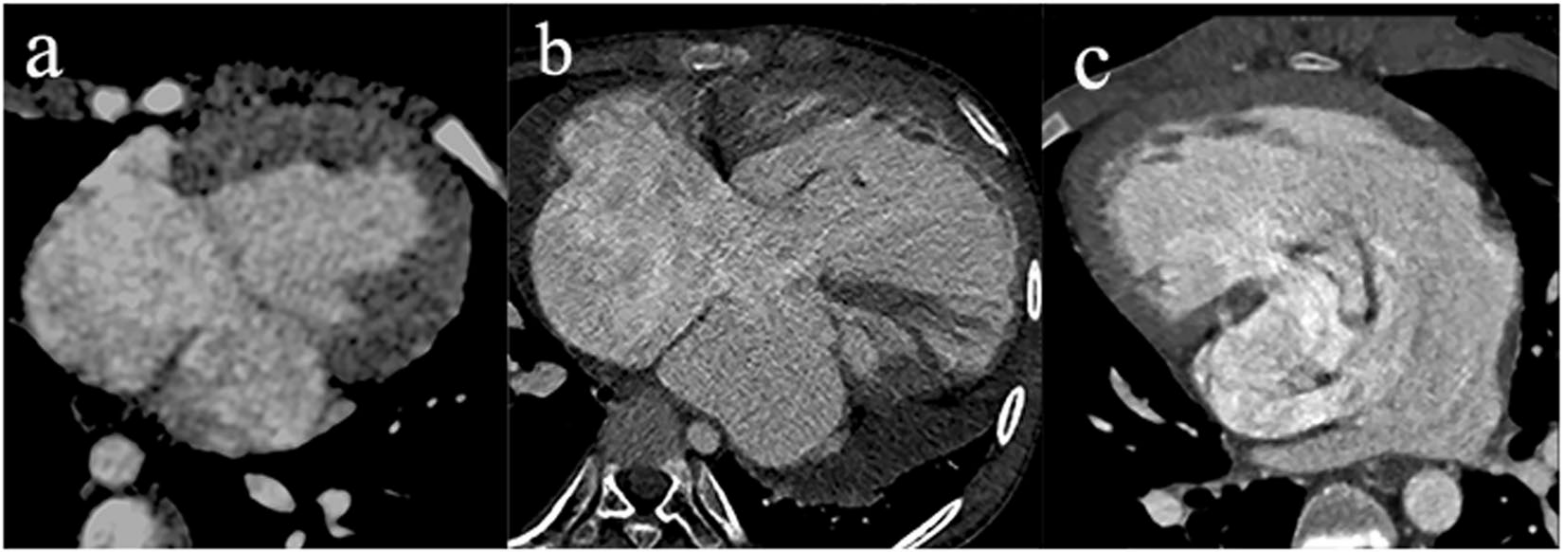
Anatomic types of SV demonstrated by DSCT. Single left ventricle. (**a**) The ventricle is characterized by relatively smooth walls, fine trabeculations and lack of septal chordal attachments of the atrioventricular valve. Single right ventricle. (**b**) The ventricle has more coarse trabeculations always accompanied with chordal attachments to the septal surface. Undifferentiated ventricle. (**c**) The ventricle has the features which are hard to divide into the two types mentioned above. DSCT indicates dual-source computed tomography; SV, single ventricle.

There were no significant differences in age, weight, height, or body surface area between the SV patients and control subjects in our study (all p > 0.05) (Table 1). The mean diameter of MPA by DSCT in patients(0.81 ± 0.36 cm) was smaller than control subjects(1.56 ± 0.18 cm) and the diameter of AAO is larger in patients(1.93 ± 0.22 cm) than control subjects(1.52 ± 0.20 cm).The stage II operation was the most common surgery conducted in SV patients (15, 48.4%). The anatomic types of SV in the 31 patients consisted of single left ventricle (n = 10, 32.3%), single right ventricle (n = 11, 35.5%), and undifferentiated ventricle (n = 10, 32.3%) (Fig. 1). After confirmation at surgery or by catheterization, the locations of MPA and AAO (n = 26) included normal arrangements (n = 3, 11.5%), right-sided aortic arch (n = 15, 57.7%), and left-sided aortic arch (n = 8, 30.8%). Three (9.7%) cases of AAO dilation, three (9.7%) of MPA atresia, 19 (61.3%) of MPA stenosis, and three (9.7%) of MPA dilation were verified among the 31 patients.

**Table 1:** Baseline characteristics.

Variable	Patients (n = 31)	Normal control (n = 40)	*P* value
Sex			
Male	18(58.1%)	22(55%)	0.80
Female	13(41.9%)	18(45%)	0.80
Age,months	50.48 ± 42.62	58.28 ± 49.14	0.49
Height,cm	92.19 ± 25.78	99.33 ± 33.55	0.33
Weigt,kg	14.36 ± 8.27	19.19 ± 12.92	0.08
BSA,m^2^	0.59 ± 0.26	0.72 ± 0.36	0.09
Heart Rate,bpm	114.42 ± 18.65	108.67 ± 20.52	0.08
Time from CT to echo,d	6 (1 to 8)	5 (1 to 6)	0.67
Time from CT to surgery or Catheterization,d	5 (1 to 4)	—	—
Diameter of MPA measured by CT,cm	0.81 ± 0.36	1.56 ± 0.18	<0.01
Diameter of AAO measured by CT,cm	1.93 ± 0.22	1.52 ± 0.20	0.01
**Anatomic type of single ventricle**			
Single left ventricle	10(32.3%)	—	—
Single right ventricle	11(35.5%)	—	—
Undifferentiated ventricle	10(32.3%)	—	—
**Surgery or Catheterization**		—	—
Stage I	4(12.9%)	—	—
Stage II	15(48.4%)	—	—
Stage III	3(9.7%)	—	—
Catheterization	17(54.8%)	—	—

Notes: BSA indicates body surface area; CT, computed tomography.

### Diagnostic accuracy for great arteries

DSCT is superior to TTE in detection of stenosis or dilation of MPA (sensitivity: DSCT 88.0% vs. TTE 80.0%; specificity: DSCT 50.0% vs. TTE 33.3%), AAO (sensitivity: DSCT 100% vs. TTE 66.7%; specificity: DSCT 92.9% vs. TTE 50.0%), and great arteries location (sensitivity: DSCT 95.7% vs. TTE 95.2%; specificity: DSCT 100% vs. TTE 100%) (Table 2).

**Table 2:** Diagnostic accuracy for great arteries.

	DSCT			Echo		
	MPA	AAO	Great arteries Location	MPA	AAO	Great arteries location
Diagnostic Values						
Sen	88.0%	100%	95.7%	80.0%	66.7%	95.2%
Spec	50.0%	92.9%	100%	33.3%	50.0%	100%
FNR	12.0%	0%	4.4%	20.0%	33.3%	4.8%
FPR	50.0%	7.1%	0%	66.7%	50.0%	0%
Agreements with established diagnostic standard						
Kappa	0.504	0.716	0.864	0.377	0.068	0.644
*P* value	0.01	<0.01	<0.01	>0.05	>0.05	<0.01

Notes: AAO indicates ascending aorta; FNR, false negative rate; FPR, false positive rate; Kappa, kappa value; MPA, main pulmonary artery; Sen, sensitivity; Spec, specificity.

The kappa values of DSCT were 0.504, 0.716, and 0.864 for evaluation of MPA, AAO, and great arteries location, respectively. The kappa values of TTE were 0.377, 0.068, and 0.644 for evaluation of MPA, AAO, and great arteries location, respectively.

### Correlation between DSCT-based artery diameter and artery pressure

In the patients undergoing catheterization, the mean values of DSCT-based diameters (n = 17) of MPA and AAO were 1.07 ± 0.41 cm and 2.19 ± 0.66 cm, respectively. Mean pressures (n = 27, 10 patients had measurements both before and after oxygen inhalation) of MPA and AAO were 18.81 ± 13.41 mmHg and 62.15 ± 10.93 mmHg, respectively. Weak correlation was found between MPA diameter and its pressure (r = 0.399, p = 0.04) and moderate correlation was found between AAO diameter and pressure (r = 0.611; p = 0.01).

### Detection of accompanying arterial malformations

**Figure 2:**
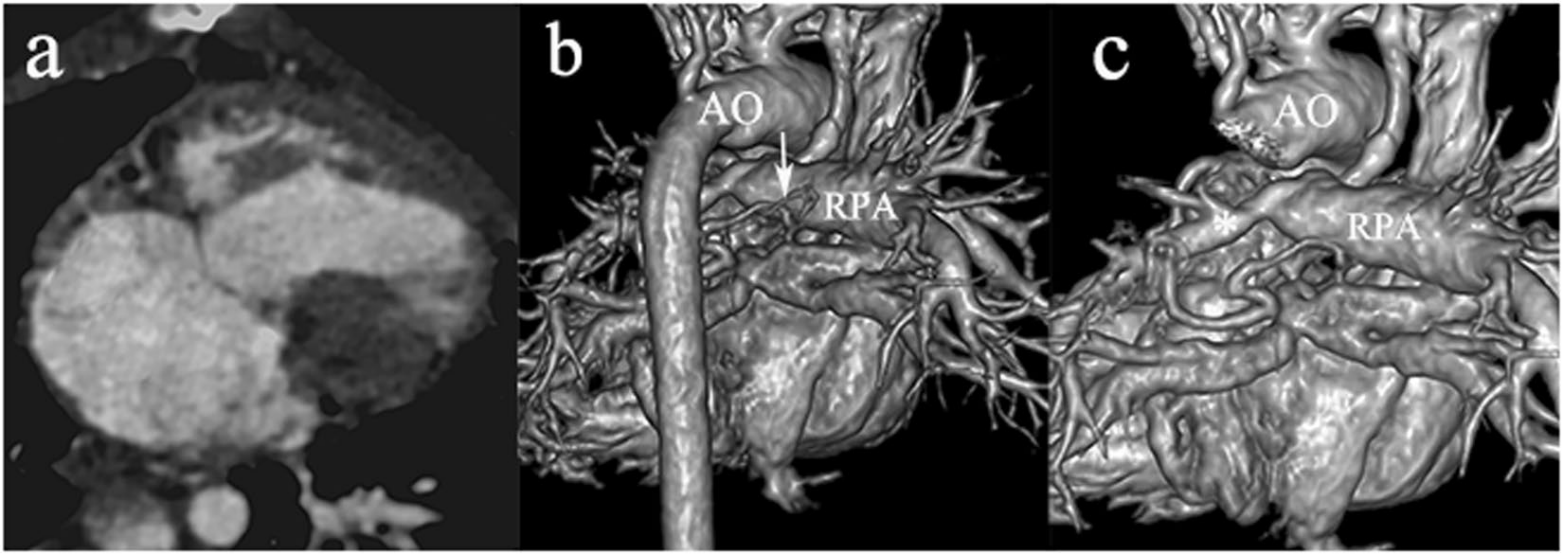
Accompanying arterial malformations detected by DSCT in a 9-year-old female with SV. (**a**) The transverse plane of univentricle of the patient. (**b**) The systemic-to-pulmonary collaterals detected by DSCT: The collaterals (arrow) arise between RPA and AO. (**c**) The PA distortion detected by DSCT: LPA (asterisk) is markedly hypoplastic. AO indicates aorta; DSCT, dual-source computed tomography; LPA, left pulmonary artery; RPA, right pulmonary artery; SV, single ventricle.

The accompanying arterial malformations detected by DSCT included patent ductus arteriosus (PDA) (n = 23, 74.2%), systemic-to-pulmonary collaterals (n = 26, 83.9%), PA distortions (n = 15, 48.4%), and coronary artery anomalies (n = 9, 29.0%) (Fig. 2).

### Radiation doses

The effective computed tomography radiation dose for patients in different age groups were 2.85 ± 2.03 mSv (4 months to 1 year), 3.12 ± 3.12 mSv (1 to 6 years), and 1.95 ± 1.92 mSv (6 to 12 years) (Table 3).

**Table 3:** Radiation dose estimation according to different age groups.

	4 months to 1 year	1 year to 6 years	6 years to 12 years
CTDvol(mGy)	10.46 ± 6.06	12.51 ± 6.09	10.99 ± 4.54
DLP(mGy.cm)	73.14 ± 52.13	119.88 ± 119.80	162.25 ± 151.60
ED(mSv)	2.85 ± 2.03	3.12 ± 3.12	1.95 ± 1.92

Notes: CTDvol indicates volume CT dose index; DLP,dose-length product; ED,effective dose.

## Discussion

SV describes a set of congenital cardiac malformations characterized by both atria draining entirely, or almost entirely, into one functionally single ventricular chamber^1^. Influenced by the concurrence of different cardiovascular malformations, the physiology and clinical features vary in different patients depending on circulatory hemodynamics and the ventricular burden. Anomaly of the great arteries is one of the key factors requiring assessment prior to surgery.

Among several image modalities for CHD assessment, DSCT has specific benefits for pediatric patients. For example, equipped with two independent tubes and detectors, DSCT increases the acquisition speed and image quality, reducing the need for general anesthesia^7^ and heart motion artifacts^8,9^. In addition, the radiation exposure is simultaneously decreased by taking several measures such as low tube voltage, ECG-based tube, and heart rhythm adaptive pitch^10^. The accuracy of DSCT for detecting intra-cardiac and extra-cardiac lesions has been well-described in various complex CHDs^4–6^. When applied to SV, we also found that DSCT gave accurate information on not only intra-cardiac structures, diagnosing the SV anatomic type, but also the extra-cardiac great arteries to aid in surgical planning.

The stage I operation for SV patients includes different procedures for different cardiovascular malformations, while information regarding the presence or absence of PA atresia, obstruction, and dilation plays a key role in procedure selection^1^. Moreover, the PA stenosis caused by preliminary interventions also needs to be corrected during stage II and III surgeries^11,12^. As previous studies have shown, TTE exhibited poor performance in PA stenosis detection due to the interference of lung tissue^3^, and computed tomography angiography was equally accurate in assessing PA anatomy compared with chatheterization and surgery^7,9,13^. Similar to these studies, our data indicated that DSCT had a better diagnostic performance than TTE in the detection of MPA stenosis or dilation, with a sensitivity of 88.0% vs. 80.0%.

Of equal importance, aortic dysplasia in SVs must be reconstructed using the Norwood procedure; and recoarctation increases the risk of mortality and morbidity and should be treated in following treatments^1,11,12^. According to earlier research^3,14^, TTE reliably evaluated aortic coarctation. We further confirmed that DSCT was more accurate than TTE in the evaluation of AAO in SV, with higher sensitivity and specificity (ranging 92.9–100%) than TTE (50.0–66.7%). However, poor agreement was observed between TTE and the reference standard in detecting AAO dilatation, but good agreement for DSCT was documented in this present study. This discrepancy might be explained by the fact that there was only dilation, with no coarctation of AAO in the recruited patients, and TTE underestimated arterial measurements and had the limitation of operator dependence^3,7^.

Clinically, the location of the great arteries is also important for preoperative artery assessment. We concluded that DSCT gave better definition in locating MPA and AAO compared with TTE. More importantly, DSCT is able to reconstruct 3D images of extra-cardiac vessels, reflecting the positional relationship of them more intuitively to surgeons.

Apart from the anatomy of the great arteries, hemodynamics, including artery pressure, are also taken into consideration before SV surgery^1,11^. In a recent meta-analysis, there was an association between the diameter ratio of MPA to AAO and PA pressure, and CT-based diameter ratio measurement may play an important role in aiding diagnosis of pulmonary hypertension^15^. Our data also revealed the correlation between the DSCT-based diameter of arteries and their pressures, and demonstrated that the diameter of an artery is an influencing factor on arterial pressure; this association also exists in SV. Thus, DSCT has the potential to offer information reflecting whether pulmonary hypertension exists in patients with SV.

The advantages of computed tomography angiography in describing arterioles have been widely accepted^8,16^. We found that DSCT was able to detect some accompanying arterial malformations of clinical importance while conducting assessment of the great arteries. PDA and coronary artery anomalies should be taken into consideration in formulating the surgical strategy^11,12,17^. Systemic-to-pulmonary collaterals may lengthen the recovery time and their detection should prompt embolization intervention^18,19^. In addition, PA distortion is an independent risk factor for patient mortality^20^. In summary, the detection of the existence of these anomalies by DSCT provides valuable information for interventional planning and outcome prediction of SV.

Our study had several limitations. First, because SV is a rare class of CHD, our single-center study is not based on a large number of patients. Further multi-center research is necessary for larger sample enrollment. Second, the pediatric population is sensitive to the diagnostic radiation exposure. In this study, we took several measures to reduce radiation exposure, which was much lower than that for catheterization. It will be possible to further decrease this dose with methods such as perspective ECG-gated scanning mode. Third, our study did not include follow-up outcomes, and these will be documented in a future study.

In conclusion, DSCT has the ability to give a more reliable assessment of the pulmonary artery and aorta than TTE which are vital in surgical planning in pediatric patients with SV, and simultaneously provides additional arterial information valuable to clinical interventions.

## Methods and Materials

### Study population

From January 2010 to November 2016, a total of 53 pediatric SV patients in our hospital were retrospectively enrolled by searching our Cardiovascular Program database, and the inclusion criteria were in accordance with the ACC/AHA 2008 Guidelines^21^. The exclusion criteria included poor image quality (n = 6, 11.3%) or incomplete clinical data (n = 16, 30.2%) and finally 31 patients (18 males, 13 females) who underwent both TTE and DSCT examinations remained. We simultaneously recruited 40 pair-matched control subjects (22 males, 18 females) suspected of having CHD by TTE but ultimately proven normal by computed tomography angiography and clinical findings.

This study was conducted with the approval of the Institutional Review Board of our hospital (No. 14-163). Guardians of all the patients gave written informed consent prior to the examinations, having been informed of potential adverse reactions to the iodinated contrast agent and radiation. The participants’ names and other HIPAA identifiers had been removed from all sections of the manuscript, including supplementary information.

### Dual-source computed tomography

All scanning was conducted on a DSCT scanner (Somatom Definition Flash; Siemens Medical Solutions, Forchheim, Germany). Subjects younger than 6 years were given a short-acting sedative (chloral hydrate at a concentration of 10%, 0.5 ml/kg) prior to the examinations. Older patients were trained to hold their breath during scanning. The acquisition parameters of the ECG-gated protocol were as follows: tube voltage of 80 kV, tube current of 100 mAs, gantry rotation time of 0.28 s, and pitch of 0.2–0.5 (selected according to the heart rate, a higher pitch was used for higher heart rates). The ECG-pulsing window was set on Auto. The scanning scope was from the thoracic inlet to 2 cm below the level of the diaphragm in the craniocaudal direction. During angiography, nonionic contrast agent (iopamidol, 370 mg/ml; Bracco, Italy) was given at a rate of 1.2–2.5 ml/s via an antecubital vein, followed by 20 ml of saline solution at the same flow rate. The injected volume was based on body weight (1.5 ml/kg). Bolus tracking was used in the region of interest (ROI) in the descending aorta with a predefined threshold of 100 HU. Image acquisition was triggered following a delay of 5 s when the ROI attenuation threshold reached 100 HU. Data processing was performed on a workstation (Syngo; Siemens Medical System, Forchheim, Germany). The images were reconstructed with a slice thickness of 0.75 mm and an increment of 0.7 mm.

### Trans-thoracic echocardiography

All patients underwent the echocardiography examination with a Philips SONOS 7500 ultrasound system (Philips Medical Systems, Bothell, WA). Established protocols for TTE, including M-mode, two-dimensional, continuous wave, and color Doppler flow imaging, were performed according to the recommendations of the American Society of Echocardiography Committee^22^. The interval between DSCT and TTE was less than 9 days.

### Image analysis

**Figure 3:**
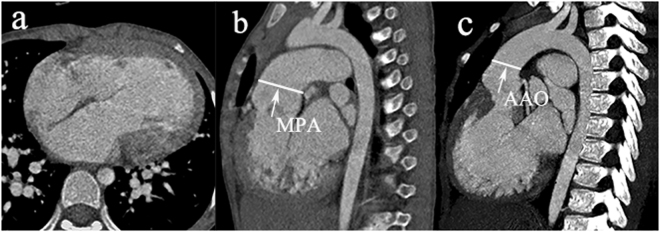
Measurements of MPA and AAO in a 9-year-old male with SV. (**a**) The transverse plane of univentricle of the patient. (**b**) The measurement of MPA is conducted 1 cm above the pulmonary arterial valve. (**c**) The measurement of AAO is conducted 1 cm above the aortic sinus. AAO indicates ascending aorta; MPA, main pulmonary artery; SV, single ventricle.

The DSCT assessments were conducted by two experienced radiologists, blinded to each other’s diagnoses, within a three-day period. The measurements of MPA and AAO diameters were at sites 1 cm above the PA valve and aortic sinus, respectively^22^ (Fig. 3). Using the mean diameters of MPA and AAO of control subjects as a contrast standard, the DSCT-based diameters of both arteries in patients were compared with the control values: MPA stenosis was defined as <75% contrast standard^3,23^ and MPA dilation was defined as Z-score (standard deviation units from the mean) >2^24^. AAO dilation and stenosis were regarded as Z-score > 2 and Z-score < −2, respectively^25^.

### Radiation dose estimation

Volume CT dose index and dose-length product were automatically displayed on the CT console after examination. The effective dose was obtained by using size-specific dose estimates^26^.

### Statistical analysis

The data were analyzed using SPSS software for Windows (version 20.0; SPSS Inc., Chicago, IL, USA). Continuous variables were expressed as mean ± standard deviations. Categorical variables were expressed as numbers and percentages (or range). Using the surgical and catheterization results as an established diagnostic standard, the diagnostic accuracy of DSCT and TTE in the evaluation of MPA, AAO, and great arteries location were calculated as sensitivity, specificity, false negative rate, and false positive rate. The diagnostic agreement of DSCT and TTE with the established diagnostic standard in the three fields mentioned above was assessed using kappa values. Correlations between the DSCT-based diameters of arteries and their pressures were analyzed by Spearman correlation coefficients. P values less than 0.05 were considered statistically significant.

### Data availability statement

The datasets generated during and analyzed during the current study are available from the corresponding author on reasonable request.
